# A method for selecting *cis*-acting regulatory sequences that respond to small molecule effectors

**DOI:** 10.1186/1471-2199-11-56

**Published:** 2010-08-10

**Authors:** Ülar Allas, Tanel Tenson

**Affiliations:** 1 Institute of Technology, University of Tartu, Nooruse 1, Tartu 50411, Estonia

## Abstract

**Background:**

Several *cis*-acting regulatory sequences functioning at the level of mRNA or nascent peptide and specifically influencing transcription or translation have been described. These regulatory elements often respond to specific chemicals.

**Results:**

We have developed a method that allows us to select *cis*-acting regulatory sequences that respond to diverse chemicals. The method is based on the β-lactamase gene containing a random sequence inserted into the beginning of the ORF. Several rounds of selection are used to isolate sequences that suppress β-lactamase expression in response to the compound under study. We have isolated sequences that respond to erythromycin, troleandomycin, chloramphenicol, meta-toluate and homoserine lactone. By introducing synonymous and non-synonymous mutations we have shown that at least in the case of erythromycin the sequences act at the peptide level. We have also tested the cross-activities of the constructs and found that in most cases the sequences respond most strongly to the compound on which they were isolated.

**Conclusions:**

Several selected peptides showed ligand-specific changes in amino acid frequencies, but no consensus motif could be identified. This is consistent with previous observations on natural *cis-*acting peptides, showing that it is often impossible to demonstrate a consensus. Applying the currently developed method on a larger scale, by selecting and comparing an extended set of sequences, might allow the sequence rules underlying the activity of *cis-*acting regulatory peptides to be identified.

## Background

There are several ways in which the translation of an mRNA can be regulated. According to the classical scenario, *trans *factors, either protein or RNA, bind to the mRNA and modulate the level of translation. More recently, regulatory mechanisms not requiring *trans*-acting protein or RNA molecules have been described. First, the nascent peptide can stall the ribosome, often in response to small molecules. This leads to the induction or repression of downstream genes [1,2]. In bacteria, nascent peptide:ribosome complexes respond to macrolide group antibiotics, chloramphenicol and tryptophan [3-6]. Secondly, the mRNA itself can recognize small molecules, leading to changes in its transcription or translation. These regulatory RNA structures are called riboswitches [7,8].

Interestingly, the nascent peptides that regulate the activity of the ribosome, in a similar manner in most cases, do not share a consensus sequence [1,4]. Finding the sequence rules is hampered by the limited number of sequences available. Although several screens for active peptide sequences have been published [9-12], a general method that allows identification of regulatory sequences responding to small molecule effectors was lacking. Here, we describe a novel method for selecting peptides capable of inhibiting translation in response to different chemicals. The method allows new regulatory elements to be constructed and might shed light on the sequence rules underlining the activities of natural regulatory peptides. Although developed for identifying small peptide sequences, the method might be applicable to the development of synthetic riboswitches.

## Results

### Rationale of selection

Expression libraries for random peptides provide a useful tool for isolating peptides with various properties [11-14]. In this study, a method was developed for selecting *cis*-acting sequences that sensitize the synthesis of a specific marker protein to externally added chemicals. We expected the sequences to exert activity at the peptide level.

We built our selection scheme upon β-lactamase. Ampicillin induces autolysis of sensitive bacteria and β-lactamase provides resistance to it. When a random peptide sequence added to the N-terminus of β-lactamase (or a nucleotide sequence inserted in front of the ORF) decreases the expression of the enzyme in response to a specific chemical, a cell bearing this plasmid becomes sensitive and lyses, releasing the plasmid into the growth medium. The sequences that specifically respond to the compound of interest can be identified in the plasmid DNA released under these conditions.

The set of compounds used for selection included the antibiotics erythromycin and chloramphenicol, which bind to the large ribosomal subunit near the peptidyl transferase center and inhibit protein synthesis [15-18]. For both these antibiotics, *cis*-acting regulatory peptides have been reported [2-4,19]. We also extended our studies to compounds that are not known to have specific peptide response sequences. Troleandomycin is structurally and functionally related to erythromycin [14], so it is highly likely to select regulatory peptide sequences. Homoserine lactones (HSL) are well-described bacterial signal molecules that are produced by many different species but not *E. coli *[20]. Meta-toluate, an aromatic compound foreign to *E. coli*, was included to increase the diversity of chemicals tested.

### Selection of *cis*-acting sequences

We prepared a plasmid library in which an SD, initiation codon and 21 random nucleotides were inserted in front of the coding sequence of the β-lactamase ORF. Transcription was controlled by the *tac *promoter [21]. Bacteria were transformed with the plasmid library and the transformants were initially grown at a low concentration of ampicillin. This selection step was necessary to eliminate clones in which β-lactamase activity had been constitutively reduced; for example, as a result of introducing a termination codon into the random sequence. Our initial library contained 10^6 ^clones, containing all four nucleotides in approximately equal amounts (21% A, 20.5% C, 25.1% G and 33.4% T). The possible number of RNA sequences coded by a random sequence of 21 nucleotides is over 10^12 ^and the number of possible heptapeptide sequences is 1.28*10^9^. Therefore only a small fraction of the sequence space was sampled in our experiments. Still, we expected that if strong sequence preference patterns are involved these would be revealed. For example, the amino acid preference patterns that we have observed for the macrolide resistance peptides [11,13,14,22] would be easily determined using the library size of the current study.

In the next step, the cells were collected, washed to remove ampicillin and transferred into fresh medium containing one of the compounds used for selection. The chemicals were used at concentrations inhibiting the growth rate by ~20% with the exception of HSL, which does not significantly inhibit cell growth. After the cells had been grown in the presence of the chemical, ampicillin was added. If the 21-nucleotide insertion (or the peptide encoded) inhibited β-lactamase synthesis, the cells became sensitive to ampicillin and were lysed. Since clones in which inhibition occurred without an externally added chemical were removed in the first selection step, the second step is expected to enrich for sequences responding to the chemical. To isolate the sequences suppressing β-lactamase production, plasmids released from the lysed cells were isolated from the medium and used to transform new bacteria. The selection steps described above were repeated for two more rounds.

In the next step, we isolated individual clones and analyzed ampicillin sensitivity in the absence and presence of the compound under study; 4-7% of the clones were more sensitive to ampicillin than the control construct in the presence of the test compound. The plasmids were isolated from these cells and the region randomized in the original library was sequenced. For every test compound, at least 10 clones were chosen for further analysis.

### Selected sequences

All the compounds used selected for sequences that sensitized the bacteria to ampicillin (Table 1, Additional file 1). The activities are reported as ratios of optical densities measured in the absence and presence of the corresponding compound. We confirmed that the sequence variations concern only the randomized region, with no PCR generated mutations in the other parts of the β-lactamase coding ORF by sequencing the plasmid from clones giving the strongest response (from six strongest clones per selection). We recognized no common RNA sequence consensus among the clones selected on any of the compounds. Similarly, no common sequence motifs were found at the peptide level. Nevertheless, the amino acid compositions of peptides selected on different compounds revealed some trends. Peptides selected on erythromycin were enriched in leucine and serine; sequences selected on chloramphenicol were enriched in glycine; troleandomycin selected for enrichment in arginine; and sequences selected on HSL were enriched in valine (Table 2).

**Table 1:** The activities of the constructs isolated as measured in three reporter systems.

**Erythromycin**		**activity in reporter system**		
**name**	**amino acid sequence**	**lactamase**	**galactosidase**	**GFP**
E1	Asp His Tyr Ser Arg Ile Val	2.02 (±0.11)	1.46 (±0.10)	ND
E2	Val Ile Gly Arg Asn Ser Phe	1.70 (±0.09)	1.85 (±0.04)	ND
E3	Gly Thr Ser Leu Gly Arg Gly	1.66 (±0.09)	1.61 (±0.08)	ND
E4	Leu Gly Ile Arg Ser Gly Tyr	1.63 (±0.11)	2.04 (±0.03)	1.62 (±0.12)
E5	Ile Ser Val Trp Leu Val Ala	1.58 (±0.16)	1.95 (±0.05)	1.55 (±0.01)
E6	Gly Trp His Leu Arg Leu Val	1.56 (±0.14)	1.95 (±0.06)	1.33 (±0.05)
E7	Val Gly Leu Phe Ala Asn Gly	1.54 (±0.12)	1.53 (±0.08)	ND
E8	Ala Ser Ser Val Ser His Leu	1.53 (±0.21)	1.73 (±0.12)	ND
E9	Phe Ile Leu Ser Ile Ser Leu	1.48 (±0.17)	1.63 (±0.06)	ND
E10	Leu Val Leu Cys Asn Leu Leu	1.46 (±0.07)	1.41 (±0.04)	ND
control	-	1.06 (±0.13)	1.26 (±0.06)	1.15 (±0.04)
**Chloramphenicol**		**activity in reporter system**		
**name**	**amino acid sequence**	**lactamase**	**galactosidase**	**GFP**
C1	Gln Glu Arg Ala Thr Gly Val	3.06 (±0.18)	2.46 (±0.06)	1.95 (±0.02)
C2	Arg Gly Gly Phe Ser Asn Glu	2.89 (±0.06)	1.73 (±0.08)	ND
C3	Glu Asn Ala Pro Ser Tyr Asp	2.62 (±0.10)	2.00 (±0.14)	ND
C4	Ser Ser Gly Ala Phe Gly Thr	2.49 (±0.04)	1.74 (±0.12)	ND
C5	Arg Val Pro Pro Arg Arg Cys	2.35 (±0.14)	2.08 (±0.09)	ND
C6	Phe Leu Glu Val Gly Cys Ser	2.32 (±0.11)	2.40 (±0.03)	3.11 (±0.02)
C7	Gly Arg Gln Val Gly Arg Val	2.14 (±0.10)	1.72 (±0.11)	ND
C8	Ser Val Cys Cys Phe Val Phe	2.01 (±0.08)	2.05 (±0.13)	ND
C9	Cys Trp Gly Ile Asp Met Ala	2.00 (±0.15)	2.17 (±0.04)	1.91 (±0.05)
C10	Lys Thr Tyr Val Gly Lys Ile	1.93 (±0.12)	1.95 (±0.07)	ND
control	-	1.41 (±0.06)	1.43 (±0.12)	1.43 (±0.08)
**Troleandomycin**		**activity in reporter system**		
**name**	**amino acid sequence**	**lactamase**	**galactosidase**	**GFP**
T1	Asn Leu Glu Ser Val Leu Ser	2.03 (±0.14)	2.36 (±0.11)	0.46 (±0.02)
T2	Arg Val Arg Asp Val Phe Tyr	1.78 (±0.05)	1.53 (±0.10)	ND
T3	Arg Gly Glu Gly Trp Gly Leu	1.67 (±0.15)	1.63 (±0.14)	ND
T4	Gly Arg Gly Arg Cys Val Ser	1.65 (±0.06)	2.14 (±0.10)	0.70 (±0.21)
T5	Ser Ala Glu Ala Phe Arg Ala	1.58 (±0.08)	1.51 (±0.07)	ND
T6	Ile Leu Met Leu Arg Arg Ser	1.56 (±0.13)	1.62 (±0.11)	ND
T7	Gly Gly Ser Ala Phe Ile Glu	1.45 (±0.13)	1.53 (±0.04)	ND
T8	Glu Cys Gln Ser Arg His Met	1.37 (±0.10)	1.67 (±0.08)	ND
T9	Ile Ser Ile Met Arg Arg Cys	1.21 (±0.08)	1.85 (±0.06)	0.81 (±0.10)
T10	Pro Ser Ala Arg Ile His Arg	1.20 (±0.11)	1.38 (±0.13)	ND
control	-	1.11 (±0.13)	1.54 (±0.03)	0.91 (±0.05)
**Meta-toluate**		**activity in reporter system**		
**name**	**amino acid sequence**	**lactamase**	**galactosidase**	**GFP**
M1	Glu Arg Thr Tyr Ile Met Tyr	2.35 (±0.13)	0.99 (±0.08)	1.34 (±0.07)
M2	Ser Trp Gly Leu Ala Ala Lys	2.24 (±0.08)	0.73 (±0.09)	1.39 (±0.06)
M3	Arg Ala Asp Arg Phe Lys Phe	2.14 (±0.04)	1.10 (±0.01)	1.86 (±0.15)
M4	Lys Tyr Gln Leu Leu Ile Cys	1.90 (±0.12)	1.21 (±0.06)	1.24 (±0.05)
M5	Gly Trp His Arg Trp Trp Tyr	1.84 (±0.06)	0.97 (±0.06)	1.26 (±0.04)
M6	Gly Lys Phe Gly Ser Ala Ser	1.81 (±0.11)	1.04 (±0.05)	1.38 (±0.12)
M7	Leu Val Val Thr Thr Leu Ile	1.80 (±0.08)	0.89 (±0.04)	1.69 (±0.15)
M8	Gly Lys Leu Tyr Arg Tyr Asp	1.75 (±0.05)	0.98 (±0.03)	1.65 (±0.13)
M9	Ile Ser Val Trp Leu Val Ala	1.67 (±0.05)	0.94 (±0.09)	0.97 (±0.05)
M10	Ser Arg Val Leu Val Ser Cys	1.64 (±0.06)	1.06 (±0.02)	2.23 (±0.22)
control	-	1.34 (±0.04)	0.89 (±0.11)	1.63 (±0.05)
**HSL**		**activity in reporter system**		
**name**	**amino acid sequence**	**lactamase**	**galactosidase**	**GFP**
H1	Thr Lys Leu Ser Pro Leu Ile	1.44 (±0.11)	ND	1.23 (±0.06)
H2	Ala Leu Pro Val Val Val Ile	1.39 (±0.05)	ND	1.49 (±0.06)
H3	Gly Ala Val Asp Val Leu Val	1.37 (±0.13)	ND	1.24 (±0.12)
H4	Asp Tyr Val Asp Cys Pro Arg	1.35 (±0.06)	ND	1.44 (±0.04)
H5	Phe Leu Cys Pro His Asp Ser	1.33 (±0.08)	ND	1.18 (±0.02)
H6	Glu Leu Phe Val Pro Tyr Cys	1.23 (±0.07)	ND	ND
H7	Lys Ala Ser Leu Asp Pro Leu	1.21 (±0.09)	ND	ND
H8	Gly Ala Phe Met Val Asp Ser	1.13 (±0.07)	ND	ND
H9	Asn Ala Ile Ala Ile Arg Arg	1.12 (±0.15)	ND	1.56 (±0.11)
H10	Asn Val Leu Ala Phe Lys Gly	1.11 (±0.10)	ND	1.26 (±0.04)
control	-	0.96 (±0.11)	ND	1.32 (±0.05)

The activity is shown as a ratio of marker gene expression measured in the absence and presence of the corresponding compound. The plasmids without 5' extensions in the marker gene coding ORFs, p177-NotI, pPOTZ-HindIII and pETGFP, were used as controls for β-lactamase, β-galactosidase and GFP activity respectively. Amino acid sequences of the peptides encoded by the randomized part of the plasmid are shown. Standard errors are indicated. ND - not determined.

**Table 2:** Number of amino acids in the randomized part of the sequences (Table 1), analyzed according to the chemical used in selection.

	erythromycin		chloramphenicol		troleandomycin		meta-toluate		HSL	
	
Amino acid	Total number	Sequences	Total number	Sequences	Total number	Sequences	Total number	Sequences	Total number	Sequences
A	3	3	4	4	5	3	5	4	7	6
C	1	1	5	4	3	3	2	2	3	3
D	1	1	2	2	1	1	2	2	6	5
E	-	-	4	4	5	5	1	1	1	1
F	3	3	5	4	3	3	3	2	4	3
G	9	5	10	7	7	3	5	4	3	3
H	3	3	-	-	2	2	1	1	1	1
I	6	5	2	2	5	4	4	4	4	3
K	-	-	2	1	-	-	5	5	3	2
L	13	8	1	1	5	3	8	6	9	7
M	-	-	1	1	3	3	1	1	1	1
N	3	3	2	2	1	1	-	-	2	2
P	-	-	3	2	1	1	-	-	6	6
Q	-	-	2	2	1	1	1	1	-	-
R	5	5	7	4	13	8	6	5	3	2
S	10	7	6	5	9	9	6	4	4	4
T	1	1	3	3	-	-	3	2	1	1
V	8	7	8	6	4	3	6	3	10	6
W	2	2	1	1	1	1	5	3	-	-
Y	2	2	2	2	1	1	6	4	2	2

"Total number" indicates the total number of amino acids in the sequence group; "Sequences" indicates the number of sequences containing the particular amino acid.

### Activity of the selected sequences tested with alternative reporter systems

The sequences isolated might respond to specific chemicals only in the presence of ampicillin or might work only if inserted in front of β-lactamase. To test for such possible context effects we fused the selected sequences to the ORFs encoding β-galactosidase and GFP. β-galactosidase expression was measured in Miller units and GFP expression was determined by flow cytometry, measuring the green fluorescence of the cells. The activities of the selected sequences are reported as ratios of reporter gene activity measured in the absence and presence of the corresponding chemical (Table 1).

Sequences selected on erythromycin or chloramphenicol responded to the corresponding compound in the context of both additional reporter genes. However, only some of the sequences selected on troleandomycin were active in the context of β-galactosidase and none worked with the GFP reporter. Only a few of the meta-toluate-responsive sequences showed weak activity with reporter systems other than the original β-lactamase. The HSL sequences were tested only in front of the GFP reporter. The activities were very weak, if significant at all.

### The sequences selected with erythromycin are active as peptides

**Figure 1:**
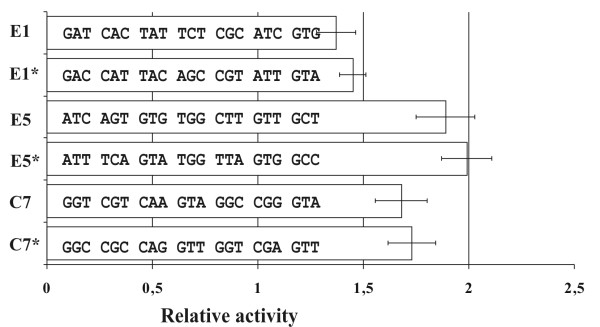
**The effects of mRNA structure on the activities of the constructs**. The activity of β-galactosidase fused with original sequences E1, E5 and C7 was compared to the activities of variants containing synonymous substitutions (marked with asterisks). The nucleotide sequences of the constructs are shown. The expression of β-galactosidase marker protein was measured 3 h after induction with IPTG in the absence and presence of the corresponding antibiotic. The activity of each construct is shown as a ratio of Miller units measured in the absence and presence of antibiotic.

There are two possible ways in which the selected sequences might affect protein synthesis. First, the sequences could affect gene expression by forming riboswitch mRNA structures, able to bind chemicals directly and regulate gene expression. Alternatively, they may be functional at the peptide level via specific interactions between the chemicals, peptides and the ribosome. To discriminate between these possibilities, we introduced synonymous mutations into one chloramphenicol and two erythromycin responsive sequences fused to β-galactosidase, changing the mRNA sequence but preserving the peptide sequence (Figure 1). When possible, the codons were substituted with synonymous ones that possess the most similar frequency of usage [23]. A β-galactosidase assay was used to compare the substituted variants with the original sequence. As shown in Figure 1, we found no significant differences between the expression levels of the initial constructs and synonymous codon-substituted variants.

**Figure 2:**
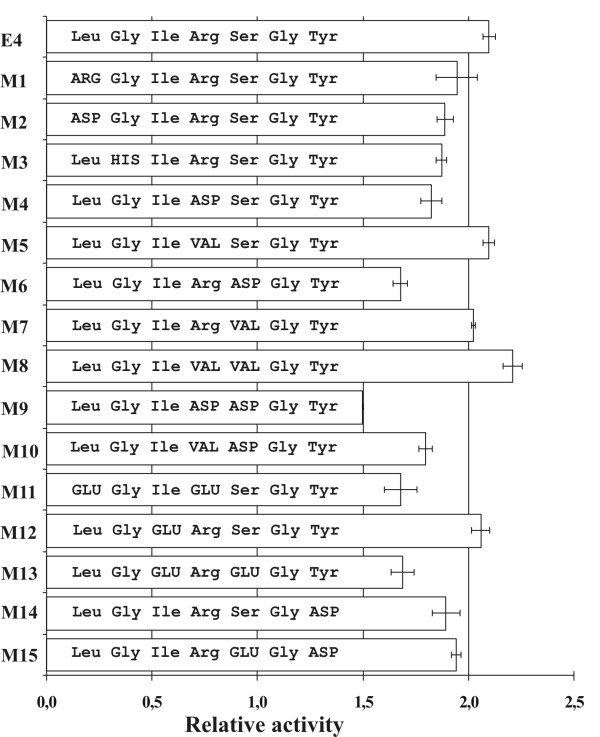
**The effects of amino acid substitutions in the E4 sequence**. Amino acid substitutions in mutants are marked in capital letters. All sequence variants were tested as β-galactosidase fusions. The expression of β-galactosidase marker protein was measured 3 h after induction with IPTG in the absence and presence of the corresponding antibiotic. The activity of each construct is shown as a ratio of Miller units measured in the absence and presence of antibiotic.

**Figure 3:**
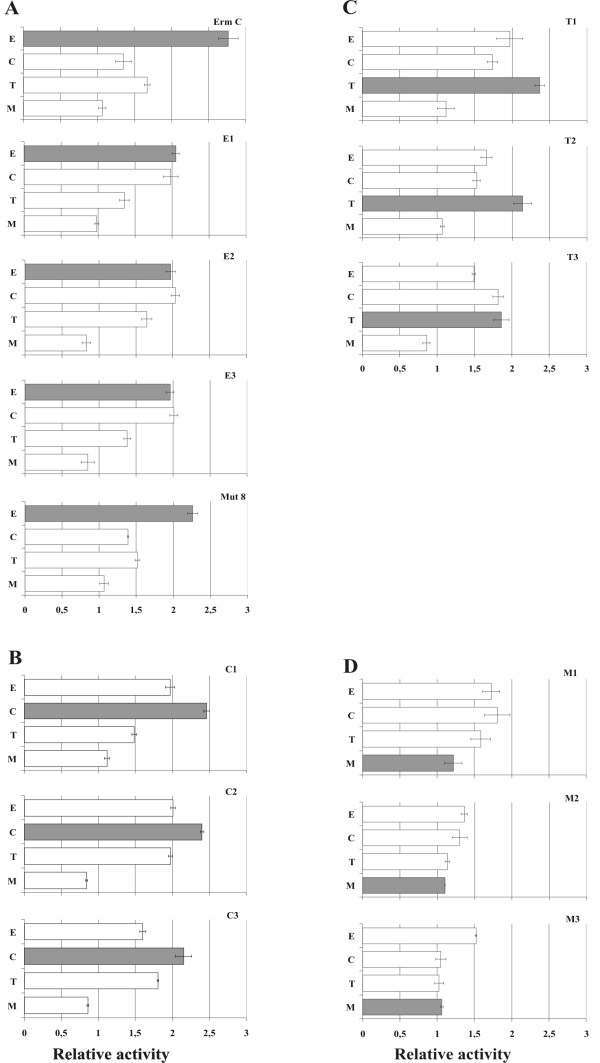
**Cross-responses of the sequences to different compounds as measured in the β-galactosidase reporter system**. The sequences originally selected on erythromycin (A), chloramphenicol (B), troleandomycin (C) and meta-toluate (D) were tested. Constructs containing ErmC leader peptide and a mutant of E4 peptide (Mut8, Figure 2) were included. The expression of β-galactosidase marker protein was measured 3 h after induction with IPTG in the absence and presence of compound (E-erythromycin, C-chloramphenicol, T-troleandomycin, M-meta-toluate). The activity of each construct is shown as a ratio of Miller units measured in the absence and presence of the compound. Gray bars indicate the activity in the presence of the compound which was used for selecting the sequence.

To test whether a selected sequence is active at the level of the translated peptide, several non-synonymous mutations changing the amino acid sequence were introduced into the erythromycin responsive element that was most active in the context of β-galactosidase. As shown in Figure 2, the single amino acid substitutions influenced the activity of the peptide. The activity was greater when the peptide was made more hydrophobic (M8). In contrast, introducing negatively charged aspartic acid residues decreased the activity (constructs M6, M9, M11, M13). It has to be noted that we do not have a negative control, a sequence that does not respond to erythromycin at all. Estimating the background from the cross-activity tests (Figure 3, described below) we conclude that erythromycin has unspecific effect in the β-galactosidase test system and the background is above one. Therefore the effect of mutations present in construct M9 could be considered being close to inactivation. The effects of mutations leading to amino acid changes were greater than those that preserved the peptide sequence (described above). We therefore conclude that the erythromycin responsive sequences act as peptides.

### The ligand specificity of the selected sequences

The specificity of the isolated peptides for the compounds used in selection was estimated in cross-inhibition experiments using the β-galactosidase and *GFP *reporter systems. For experiments with the β-galactosidase reporter system, we chose three peptides from each group (excluding the sequences selected with HSL) that showed the strongest inhibition in response to their compound of selection. We also tested the ErmC regulatory peptide, known from the literature to respond to erythromycin, and a mutant variant of one of the selected peptides with increased activity. For the GFP reporter, three sequences of each class were tested, including those selected with HSL.

**Figure 4:**
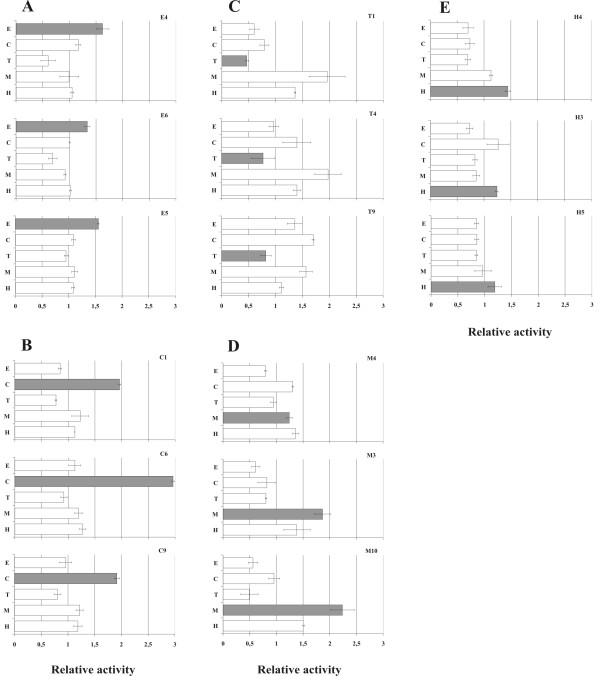
**Cross-responses of the sequences to different compounds as measured using the GFP reporter system**. The sequences originally selected on erythromycin (A), chloramphenicol (B), troleandomycin (C), meta-toluate (D) and homoserine lactone (E) were tested. The expression of GFP marker protein was measured 3 h after induction with IPTG in the absence and presence of the compound (E-erythromycin, C-chloramphenicol, T-troleandomycin, M-meta-toluate, H-homoserine lactone). The activity of each construct is shown as a ratio of GFP fluorescence measured in the absence and presence of the compound. Gray bars indicate the activity in the presence of the compound which was used for selecting the sequence.

As expected, the ErmC control sequence responded to erythromycin. The effect was in the same order of magnitude as the activities of the sequences selected in the current study. In the β-galactosidase context, all three sequences of the erythromycin group responded to both erythromycin and chloramphenicol (Figure 3, panel A). In the GFP test system, there was a specific response only to erythromycin (Figure 4, panel A). This observation shows that the reporter context influences both the magnitude and specificity of the response.

The chloramphenicol sequences responded most strongly to chloramphenicol in both reporter systems (Figure 3, panel B and Figure 4, panel B). Two of the troleandomycin sequences with the strongest effects on β-lactamase expression responded to the compound when tested with the β-galactosidase system (Figure 3, panel C). Surprisingly, these sequences did not inhibit GFP synthesis in a troleandomycin-dependent manner. In fact, addition of troleandomycin led to increased GFP synthesis (Figure 4, panel C). This shows again that the activity of a selected sequence is strongly influenced by the reporter. Moreover, some of the meta-toluate sequences seemed to have a specific effect when tested with GFP but not with β-galactosidase (Figure 3, panel D and Figure 4, panel D). One of the three HSL sequences seemed to have specific activity when tested in front of GFP. In conclusion, most of the selected peptides are specific for the compound that was used in the selection, although the effect depends strongly on the reporter system.

## Discussion

*Cis*-acting small peptides causing ribosome stalling in response to specific small molecules - erythromycin, chloramphenicol, tryptophan, arginine and polyamines - have been described previously [2-6,24-26]. In all these systems the ribosome is stalled on the mRNA in a manner depending on the peptide and the inducing chemical. It has been suggested that the stalling is initiated by a specific interaction between the chemical and the nascent peptide in the ribosome exit tunnel [1,19]. In several cases it has been shown that stalling occurs before complete translation of the ORF, indicating that it does not require the termination codon. Moreover, an arginine response peptide works as the N-terminal fusion of a reporter protein [27]. This encouraged us in designing our library to express proteins with random N-terminal sequences that could potentially regulate translation.

We have developed a method that allows us to screen for synthetic regulatory sequences responding to chemical compounds. A method of this kind should include positive selection for clones responding to the chemical and negative selection for removing clones with constitutive responses. This was achieved by using a plasmid with β-lactamase that causes ampicillin resistance. A random sequence was inserted into the beginning of the ORF and selection was carried out using three antibiotics that target the ribosome (erythromycin, troleandomycin and chloramphenicol [15-18]), one bacterial signal molecule (HSL [20]), and a non-biological aromatic compound (meta-toluate). It has to be noted that to exert its activity, β-lactamase has to be exported into the periplasm. The export signal is in the N-terminus of the protein [28,29]. In all our constructs the natural export sequence follows the randomized part. It has been shown that the export sequence can withstand many mutations without complete loss of β-lactamase activity [30]. We expect that fast majority of the library constructs can support β-lactamase export as the frequency of constructs not able to grow in the presence of ampicillin (around 30%) corresponds to the expected frequency of termination codons in the randomized part of the sequence. Still, the possibility remains that constructs are selected were the small chemicals under study suppress β-lactamase export in the peptide sequence dependent manner. These effects should not be present when tested in the context of the alternative reporter proteins. To study this possibility, the selected sequences were tested with two additional reporters, β-galactosidase and GFP. The erythromycin and chloramphenicol sequences retained activity in both these reporter systems. Surprisingly, only few of the troleandomycin sequences retained activity in the β-galactosidase system and all the sequences tested with GFP were inactive. This shows that the reporter context modulates the effect of the regulatory sequence to a large extent. The context effect points to the inherent limitation of the selection system described here and other similar systems that might be constructed; it is highly probable that some active sequences will be missed because they do not work in the context of the reporter chosen.

Our library design created a randomized part of the mRNA, leading to the production of a peptide library. The selected sequences may therefore act at the level of mRNA or peptide. To discriminate between these possibilities, one chloramphenicol and two erythromycin responsive sequences were subjected to mutational analysis. Changing the mRNA structure by introducing synonymous mutations did not change the activities of the sequences; in contrast, several mutations led to amino acid substitutions that changed the activities of the erythromycin sequences. This indicates that the activity is mediated by the protein sequence. Although we have not performed similar experiments on the other sequence classes selected, we consider it probable that these sequences also act at the peptide level. It is also possible that the N-terminal sequences do not influence the synthesis of the reporter protein but regulate the stability or activity of the reporter. To test this possibility, the erythromycin responsive sequences E4, E5 and the chloramphenicol responsive sequence C6 were studied with the GFP reporter. Transcription was induced with IPTG, followed by removal of the inducer by changing the growth medium. All cultures were divided into three aliquots. Erythromycin was added to the first aliquot, chloramphenicol to the second aliquot and the third aliquot was serving as a control. As the inducer was removed, the total fluorescence of the control culture remained constant. Addition of either antibiotic did not cause reduction of the total GFP fluorescence of the culture (data not shown) leading to the conclusion that the compounds do not influence the activity or stability of the mature reporter protein. This indicates that the sequences act during synthesis of the reporter protein suggsting a regulatory interaction in the ribosome.

In the current work we present the data as ratios of reporter gene activities measured in the absence and presence of the effector chemical. In the absence of effect, this ratio is expected to be one. The largest induction levels we found were around 2.5 fold. This is small in comparison to the reported effects of regulatory nascent peptides [26,31]. In our system, the previously described ErmC leader peptide sequence responding to erythromycin causes an effect similar in magnitude to the sequences we selected from the random library. The difference between the ErmC activity in its natural context and expressed from our selection construct might be attributed to the special arrangements of ORFs coding for natural regulatory *cis*-acting peptides. The natural bacterial regulatory ORFs are upstream of the main ORFs, the translation initiation site of the main ORF being trapped within a strong mRNA secondary structure. In response to a small chemical, erythromycin in the case of ErmC, the ribosome is stalled on the upstream ORF causing melting of the RNA secondary structure and opening of the translation initiation region of the main ORF, leading to synthesis of the enzyme responsible for erythromycin resistance [3,32]. This switching of mRNA structure might amplify the effect of ribosome stalling. The similarity in magnitude of the effects caused by the ErmC and sequences selected from the library suggests that although the observed effects are relatively small, the system might be useful for elucidating the sequence rules responsible for the functional interactions between nascent peptide chains, ribosomes and the inducing chemicals that operate in natural systems.

We have observed previously that the expression of specific small peptides can cause resistance against macrolide antibiotics, including erythromycin [11,13,14,22,33]. Macrolide antibiotics bind to the nascent peptide exit tunnel, stop growth of the polypeptide chain and cause dissociation of peptidyl-tRNA from the ribosome [15,17,34]. The resistance peptides act as a "bottle brush" causing release of the drugs from the ribosome [35]. The erythromycin resistance peptides have to be between four and six amino acids long [11], the drug being ejected during the termination event [35]. If the termination codon is replaced by a sense codon, the ribosome is stalled on the mRNA. We have speculated previously that the erythromycin resistance peptides are mechanistically related to the Erm leader peptides, the difference between possible outcomes (either drug ejection or ribosome stalling) being determined by the location of the termination codon [35]. We have used a library expressing random pentapeptides to select for sequences conferring resistance to different macrolides [11,13,14]. Interestingly, leucine is common in erythromycin resistance peptides, in line with our finding that leucine-rich sequences are selected when erythromycin is used as effector compound. Similarly, peptides conferring resistance to oleandomycin, a macrolide very similar to troleandomycin, are positively charged, in line with the enrichment with arginine observed in the current study. These observations again suggest that the mechanisms of peptide-mediated macrolide resistance and nascent peptide-mediated regulation of gene expression are similar.

Comparing the sequences selected with one chemical, we can see enrichment with certain amino acids but cannot define a consensus sequence. This is in line with the sequence conservation of natural *cis*-acting regulatory peptides, in which a consensus sequence is often hard to define. Therefore, it is currently very hard or impossible to find the ORFs encoding *cis*-acting regulatory peptides by comparing genomic sequences. Our currently developed method might be employed to investigate these issues by screening a large number of sequences, which would allow more complex sequence features than a simple consensus to be defined. The recent observation that small ORFs, widespread in all genomes, are translated more frequently than previously expected underlines the importance of these studies [36].

## Conclusion

A method for selecting *cis-*acting regulatory sequences has been developed. Although the magnitude of the effects is smaller than in the natural context, the system could be used for elucidating the sequence rules underlying natural regulatory systems.

## Methods

### Antibiotics, chemicals and bacterial strains

Erythromycin and chloramphenicol were from Amresco and Applichem respectively. Troleandomycin, meta-toluate and ONPG (ortho-nitrophenyl-β-galactoside) were from Sigma-Aldrich. HSL (N-(3-oxohexanoyl)-L-homoserine lactone) was prepared as described in [37]. *E. coli *NovaXG Zappers™ (Novagen) [*mcr*A Δ*(mcrC-mrr) endA1 recA1 *φ*80dlacZ*Δ*M15 *Δ*lacX74 araD139 *Δ*(ara-leu)7697galU galK rpsL nupG *λ*-tonA *F'[*lacIq *Tn*10*] (TetR)] electrocompetent cells were used for electroporation of the library plasmids. In all other experiments, DH5α *[fhuA2 Δ(argF-β-galactosidase)U169 phoA glnV44 Φ80 Δ(β-galactosidase)M15 gyrA96 recA1 relA1 endA1 thi-1 hsdR17] *strain was used.

### Construction of the library

The plasmid library containing seven random codons in front of the *β-lactamase *ORF was prepared following a two-step procedure:

(1) The vector pACYC177 (Fermentas) was amplified using the oligonucleotides pACYC177-1 (bearing a Shine-Dalgarno sequence) and pACYC177-2 (containing mutated tac promoter) (Additional file 2). In the same PCR reaction, NotI restriction sites were introduced at both ends of the PCR fragment. After restriction with NotI, complementary cohesive ends of the PCR fragment were ligated to form a circular DNA molecule. The plasmid obtained, p177-NotI, contains a NotI site between the *lac *operator sequence and the initiator codon of the *β-lactamase *ORF.

(2) To prepare a plasmid expression library, p177-NotI was amplified using oligonucleotides p177-BamHI and p177-BamHI2 (Additional file 2), each containing a BamHI site at the 5' end. p177-BamHI2 contained a DNA segment corresponding to the initiator codon AUG and 21 random nucleotides in front of a region complementary to the 5' end of the β-lactamase-encoding ORF. The PCR fragment was restricted with BamHI and its ends were ligated together, thus creating a plasmid library pAC-Lib. Since pAC-Lib does not contain the NotI site, the ligation mixture was incubated with NotI after inactivation of T4 DNA ligase to ensure that the library contained no traces of p177-NotI.

### Selection of active sequences

NovaXG Zappers electrocompetent cells were electroporated with the plasmid library. A 200 μl aliquot of cells was plated on a solid medium containing 25 μl/ml kanamycin to determine the efficiency of transformation. Electroporated cells were grown at 37°C in 50 ml LB medium containing 50 μl/ml ampicillin and 25 μl/ml kanamycin with shaking until the OD_600 _reached 1. The culture was then diluted to OD_600 _0.3 and grown to OD 1 in the presence of the same antibiotics. Cells were collected by centrifugation and washed with LB medium to remove the traces of ampicillin. The washed cells were used to prepare a new dilution (OD 0.3) in 50 ml LB containing kanamycin (25 μl/ml). At this point, the selecting substance was added at a concentration known to inhibit cell growth by ~20%, with the exception of homoserine lactone that does not inhibit cell growth (30 μg/ml erythromycin, 1 μg/ml chloramphenicol, 300 μg/ml trolenadomycin, 15 mM meta-toluate or 1 mM homoserine lactone). The inhibitory concentrations were determined as described in [38]. After growing the cells for 3 h, the culture was diluted to OD_600 _0.1 and grown under the same conditions as before, using the same selecting substance. When the OD_600 _reached 0.6, ampicillin was added to a final concentration of 400 μl/ml. After the cells had been grown for another hour, the plasmid was isolated from the medium: the cells were removed by centrifugation and DNA was precipitated by adding NaCl to a final concentration of 200 mM followed by 0.7 volumes of isopropanol. After centrifugation at 10,000 × g for 15 min, the DNA was dissolved in water. The isolated plasmids were used in further transformations. The selection procedure was repeated three times.

In the next phase of selection, the plasmids isolated from the liquid media were used to transform DH5α competent cells. The colonies obtained were tested for sensitivity to the selecting substance of interest in the presence of ampicillin. Cells were grown in 2 ml LB medium overnight, then the culture was diluted to OD 0.1 and transferred to a 96-well microtiter plate in duplicate. Ampicillin was added to a final concentration of 100 μl/ml to both samples; the selecting substance was added, at the concentration that inhibited cell growth by ~20%, to the test sample only. A culture carrying p177-NotI plasmid was used as reference. Samples were grown at 37°C with shaking. The OD of the cultures was recorded after 5 h. In every pair of wells, the OD_600 _determined in the absence of the selecting compound was divided into that measured in the presence of the compound. Clones that were more sensitive than the control plasmid to the test compound were subjected to the next round of testing. Overnight cultures were used to prepare a series of samples, each containing 100 μl/ml ampicillin and a defined concentration of test compound. The concentrations of test compounds were selected to cover a range from zero inhibition to twice the concentration used in the first phase of selection. The samples, diluted to OD_600 _0.1, were transferred to 96-well microtiter plates. After 5 h growth, the OD_600 _was measured and inhibition curves were calculated.

### Cloning and mutagenesis

Sequences corresponding to the selected peptides were fused to both *β-galactosidase *and *GFP *ORFs. In the first case, a HindIII linker was inserted into the BamHI site of plasmid pPOTZ, which carries a gene for β-galactosidase and confers resistance to ampicillin (the plasmid is a derivative of pPOT1 [33]; sequence provided as Additional file 3). The plasmid obtained, pPOTZ-HindIII, was partially cut with KpnI. A 7080 bp DNA fragment purified from an agarose gel was subsequently cut with HindIII, followed by purification of a 7006 bp fragment. This fragment was ligated to a HindIII/KpnI-digested PCR product that had been amplified from the library plasmid using primers β-galactosidase-KpnI and β-galactosidase-HindIII (Additional file 2) and contained sequences for the *lac *operator, *tac *promoter and the peptide tested. To fuse the selected peptide sequences to GFP, the oligonucleotides GFP Nhe linker 1 and GFP Nhe linker 2 (Additional file 2) were annealed, thus forming a linker, which was inserted at the BamHI site of plasmid pETGFP [39]. A 55 bp fragment containing SD and the sequence coding for the selected peptide was amplified by PCR from pPOTZ-based constructs using the primers GFP cloning 1 and GFP cloning 2 (Additional file 2). The amplified fragments were ligated between the NheI and BamHI sites of the modified pETGFP.

In some experiments, the selected peptide sequences fused to *β-galactosidase *in pPOTZ were mutated. This was achieved by PCR, using a long oligonucleotide containing sequences complementary to regions located upstream and downstream of the mutated sequence (Mut 1 to Mut10, Orf 2903-24, Orf 1804-16 and Orf 2501-02 in Additional file 2). The reverse oligonucleotide primer Orf-ClaI containing the ClaI site was complementary to the region inside *β-galactosidase*. The amplified fragment was restricted with BamHI/ClaI and inserted into the pPOTZ-Lib vector, from which the BamHI/ClaI fragment had been previously detached.

### β-galactosidase assay

β-galactosidase was assayed by a modification of Miller's method [40]. Cells transformed with a plasmid expressing the protein of interest were grown overnight at 37°C in LB medium containing 100 μg/ml ampicillin. The cultures were diluted with fresh medium to OD_600 _0.05 in duplicate. IPTG (final concentration 1 mM) and ampicillin (100 μg/ml) were added to both replicate samples. Erythromycin, chloramphenicol, troleandomycin or meta-toluate was added to the first at a concentration that inhibited cell growth by ~20%; there were no additions to the second. Samples for the assay were collected 3 h after induction of β-galactosidase expression. The OD_600 _of the cultures was recorded. Aliquots (50 μl) were removed from the samples and added to a mixture containing 350 μl Z-buffer [40], 5 μl chloroform and 10 μl 0.1% SDS. After vortexing, the reaction tubes were incubated for 5 min at 30°C, then 80 μl 4 mg/ml ONPG was added and the reaction mixture was incubated for 20 min at room temperature. The reaction was stopped by adding 200 μl 1M Na_2_CO_3 _and the tubes were centrifuged at 13,000 rpm for 2 min. The OD of the supernatant was measured at 420 nm and 550 nm. Miller units were calculated using the following formula:

The ability of the selected sequences to inhibit translation in the presence of the given compound was estimated as a ratio of Miller units calculated in the absence and presence of the compound.

### Flow cytometry

For flow cytometry, the cells were grown as for the β-galactosidase assay, in a medium containing kanamycin (25 μg/ml) instead of ampicillin. Samples from growing cultures collected 3 h after the induction of GFP expression were diluted with PBS and transferred to 96-well microtiter plates. To measure GFP expression levels, a flow cytometer LSR II and High Throughput Sampler (Becton-Dickinson Bioscience) were used with a laser beam maximum at 488 nm. At least 50,000 counts were acquired for each sample.

## Authors' contributions

TT designed the study, ÜA performed the experiments, and both authors participated in the data analysis and drafting of the manuscript.

## Supplementary Material

Additional file 1 **Sequences of the RNAs coded by the randomized regions of the plasmids isolated in the selection**. Click here for file

Additional file 2 **Oligonucleotides used in the study**. Click here for file

Additional file 3 **Sequence of the plasmid pPOTZ**. Click here for file
