# Supplementary material for: A method for selecting cis-acting regulatory sequences that respond to small molecule effectors
Source: BMC Mol Biol. 2010 Aug 10;11:56. doi: 10.1186/1471-2199-11-56 (PMC2928234; doi:10.1186/1471-2199-11-56)
Supplement: Additional file 1 — Sequences of the RNAs coded by the randomized regions of the plasmids isolated in the selection. [file 1471-2199-11-56-S1.DOC]

**Additional file 1.** Sequences of the RNAs coded by the randomized regions of the plasmids isolated in the selection.

| **Erythromycin** | |
| --- | --- |
| **name** | **RNA sequence** |
| E1 | GAUCACUAUUCUCGCAUCGUG |
| E2 | GUGAUUGGUCGGAAUAGUUUU |
| E3 | GGGACCAGUCUAGGAAGAGGG |
| E4 | UUAGGUAUAAGGUCAGGGUAC |
| E5 | AUCAGUGUGUGGCUUGUUGCU |
| E6 | GGGUGGCAUUUACGACUUGUA |
| E7 | GUGGGCUUGUUUGCGAAUGGU |
| E8 | GCCUCGAGUGUUAGUCACCUU |
| E9 | UUUAUUUUGUCUAUUUCCCUG |
| E10 | CUCGUCUUAUGUAACUUGCUG |
|  |  |
| **Chloramphenicol** | |
| **name** | **RNA sequence** |
| C1 | CAGGAGCGCGCGACUGGCGUU |
| C2 | CGCGGAGGAUUUAGCAAUGAA |
| C3 | GAAAAUGCACCCUCUUACGAC |
| C4 | UCUUCCGGAGCGUUUGGCACA |
| C5 | CGCGUGCCCCCUCGUCGUUGU |
| C6 | UUCCUAGAGGUGGGAUGUUCG |
| C7 | GGUCGUCAAGUAGGCCGGGUA |
| C8 | AGUGUAUGCUGUUUUGUGUUU |
| C9 | UGUUGGGGGAUUGAUAUGGCG |
| C10 | AAAACGUAUGUUGGAAAGAUU |
|  |  |
| **TROLEANDOMYCIN** | |
| **name** | **RNA sequence** |
| T1 | AAUUUAGAAUCAGUUCUUUCA |
| T2 | AGGGUGCGUGAUGUAUUCUAU |
| T3 | CGAGGAGAAGGCUGGGGGCUU |
| T4 | GGCAGGGGGAGGUGUGUAUCC |
| T5 | AGCGCGCAGGCCUUCCGAGCU |
| T6 | AUUUUAAUGCUGAGAAGAAGC |
| T7 | GGAGGAUCGGCAUUUAUCGAA |
| T8 | GAGUGCCAGUCGCGUCAUAUG |
| T9 | AUAUCGAUUAUGAGGAGGUGU |
| T10 | CCCAGUGCCAGAAUCCAUAGA |
|  |  |
| **Meta-toluate** | |
| **name** | **RNA sequence** |
| M1 | GAACGUACAUACAUUAUGUAU |
| M2 | UCGUGGGGUCUCGCUGCAAAA |
| M3 | CGAGCCGAUAGAUUUAAGUUC |
| M4 | AAGUAUCAACUUUUAAUCUGC |
| M5 | GGCUGGCACCGCUGGUGGUAC |
| M6 | GGGAAACUGUACCGUUACGAC |
| M7 | CUUGUGGUAACUACCUUGAUU |
| M8 | GGAAAGUUUGGUAGCGCUAGC |
| M9 | AUCAGUGUGUGGCUUGUUGCU |
| M10 | AGUAGGGUCCUGGUUUCGUGU |
|  |  |
| **HSL** |  |
| **name** | **RNA sequence** |
| H1 | ACAAAACUUUCUCCUUUGAUC |
| H2 | GCAUUGCCUGUUGUUGUAAUU |
| H3 | GGCGCAGUUGACGUCUUGGUG |
| H4 | GAUUAUGUAGAUUGCCCAAGG |
| H5 | UUCUUAUGUCCUCAUGAUUCG |
| H6 | GAAUUGUUUGUUCCGUAUUGU |
| H7 | AAGGCGUCCUUGGACCCCUUA |
| H8 | GGCGCAUUCAUGGUAGAUUCG |
| H9 | GACGCUAUUGCUAUUAGACGA |
| H10 | AACGUUCUUGCGUUUAAGGGU |
