# Supplementary material for: A method for selecting cis-acting regulatory sequences that respond to small molecule effectors
Source: BMC Mol Biol. 2010 Aug 10;11:56. doi: 10.1186/1471-2199-11-56 (PMC2928234; doi:10.1186/1471-2199-11-56)
Supplement: Additional file 2 — Oligonucleotides used in the study. [file 1471-2199-11-56-S2.DOC]

**Additional file 2.** Oligonucleotides used in the study.

| **Name** | **Sequence** |
| --- | --- |
| Mut 1 | 5’- GC**GGATCC**GAAAAAGGAAGAGTATG CGCGGTATAAGGTCAGGGTAC  ATGAGTATTCAACATTTAA (BamHI site bolded, mutation underlined) |
| Mut 2 | 5’- GC**GGATCC**GAAAAAGGAAGAGTATG GATGGTATAAGGTCAGGGTAC  ATGAGTATTCAACATTTAA (BamHI site bolded, mutation underlined) |
| Mut 3 | 5’- GC**GGATCC**GAAAAAGGAAGAGTATG TTACACATAAGGTCAGGGTAC  ATGAGTATTCAACATTTAA (BamHI site bolded, mutation colored) |
| Mut 4 | 5’- GC**GGATCC**GAAAAAGGAAGAGTATG TTAGGTATAGATTCAGGGTAC  ATGAGTATTCAACATTTAA (BamHI site bolded, mutation underlined) |
| Mut 5 | 5’- GC**GGATCC**GAAAAAGGAAGAGTATG TTAGGTATAGTGTCAGGGTAC  ATGAGTATTCAACATTTAA (BamHI site bolded, mutation underlined) |
| Mut 6 | 5’- GC**GGATCC**GAAAAAGGAAGAGTATG TTAGGTATAAGGGATGGGTAC  ATGAGTATTCAACATTTAA (BamHI site bolded, mutation underlined) |
| Mut 7 | 5’- GC**GGATCC**GAAAAAGGAAGAGTATG TTAGGTATAAGGGTGGGGTAC  ATGAGTATTCAACATTTAA (BamHI site bolded, mutation underlined) |
| Mut 8 | 5’- GC**GGATCC**GAAAAAGGAAGAGTATG TTAGGTATAGTGGTGGGGTAC  ATGAGTATTCAACATTTAA (BamHI site bolded, mutation underlined) |
| Mut 9 | 5’- GC**GGATCC**GAAAAAGGAAGAGTATG TTAGGTATAGATGATGGGTAC  ATGAGTATTCAACATTTAA (BamHI site bolded, mutation underlined) |
| Mut 10 | 5’- GC**GGATCC**GAAAAAGGAAGAGTATG TTAGGTATAGTGGATGGGTAC  ATGAGTATTCAACATTTAA (BamHI site bolded, mutation underlined) |
| ErmC | 5’- GC**GGATCC**GAAAAAGGAAGAGTATG TTTAGTATTTTTGTAATCAGC  ATGAGTATTCAACATTTAA (BamHI site bolded, mutation underlined) |
| Orf 2903-24 | 5’- GC**GGATCC**GAAAAAGGAAGAGTATG ATTTCAGTATGGTTAGTGGCC  ATGAGTATTCAACATTTAA (BamHI site bolded, mutation underlined) |
| Orf 1804-16 | 5’- GC**GGATCC**GAAAAAGGAAGAGTATG GACCATTACAGCCGTATTGTA  ATGAGTATTCAACATTTAA (BamHI site bolded, mutation underlined) |
| Orf 2501-02 | 5’- GC**GGATCC**GAAAAAGGAAGAGTATG GGCCGCCAGGTTGGTCGAGTT  ATGAGTATTCAACATTTAA (BamHI site bolded, mutation underlined) |
| Orf-ClaI | 5’- TGTC**ATCGAT**AATTTCACCGCCGAAAGGCGC (ClaI site bolded) |
| pACYC177 | 5’- CG**CGCGGCCG**CGAAAAAGGAAGAGTATGGA (NotI site bolded) |
| pACYC177-2 | 5’- CG**GCGGCCGC**AATTGTTATCCGCTC ACAATT CCACACATTATACGAGCCGAT TAATTGTCAAGAATTCCCAA TTCTGATTAGAAAAACTCATCGAG (NotI site bolded, tac promoter underlined) |
| p177-BamHI | 5’- GGCGC**GGATCC**AATTGTTATCCGCTCACAATTCCACAC  (BamHI site bolded) |
| p177-BamHI2 | 5’- CGCCG**GGATCC**GAAAAAGGAAGAGTATG (N)_21_ ATGAGTATTCAACATTTCCGTGTCGCCCT (BamHI site bolded) |
| GFP Nhe linker 1 | 5’- GATCCTTTTTTTAACAATTAAGGAG**GCTAGC**ACCGAGTTGGTC (NheI site bolded) |
| GFP Nhe linker 2 | 5’- GATCGACCAACTCGGT**GCTAGC**CTCCTTAATTGTTAAAAAAAG (NheI site bolded) |
| GFP cloning 1 | 5’- GC**GCTAGC**GAAAAAGGAAGAGTATG (NheI site bolded) |
| GFP cloning 2 | 5’- CG**GGATCC**GAAATGTTGAATACTCAT (BamHI site bolded) |
| HindIII linker for pPOTZ | 5’- GATCTCAAGCTTGA |
| LacZ-KpnI | 5’- CGC**GGTACC**CATTACAGAAACGGCT (KpnI site bolded) |
| LacZ-HindIII | 5’- GCG**AAGCTT**AAATGTTGAATACTCAT (HindIII site bolded) |
